# Platelet-Derived Stromal Cell-Derived Factor-1 Is Required for the Transformation of Circulating Monocytes into Multipotential Cells

**DOI:** 10.1371/journal.pone.0074246

**Published:** 2013-09-16

**Authors:** Noriyuki Seta, Yuka Okazaki, Hiroshi Miyazaki, Takashi Kato, Masataka Kuwana

**Affiliations:** 1 Division of Rheumatology, Department of Internal Medicine, Keio University School of Medicine, Tokyo, Japan; 2 Innovative Drug Research Laboratories, Research Division, Kyowa Hakko Kirin Co., Ltd., Takasaki, Japan; 3 Department of Biology, School of Education, Waseda University, Tokyo, Japan; University of Pittsburgh, United States of America

## Abstract

**Background:**

We previously described a primitive cell population derived from human circulating CD14^+^ monocytes, named monocyte-derived multipotential cells (MOMCs), which are capable of differentiating into mesenchymal and endothelial lineages. To generate MOMCs *in vitro,* monocytes are required to bind to fibronectin and be exposed to soluble factor(s) derived from circulating CD14^−^ cells. The present study was conducted to identify factors that induce MOMC differentiation.

**Methods:**

We cultured CD14^+^ monocytes on fibronectin in the presence or absence of platelets, CD14^−^ peripheral blood mononuclear cells, platelet-conditioned medium, or candidate MOMC differentiation factors. The transformation of monocytes into MOMCs was assessed by the presence of spindle-shaped adherent cells, CD34 expression, and the potential to differentiate *in vitro* into mesenchymal and endothelial lineages.

**Results:**

The presence of platelets or platelet-conditioned medium was required to generate MOMCs from monocytes. A screening of candidate platelet-derived soluble factors identified stromal cell-derived factor (SDF)-1 as a requirement for generating MOMCs. Blocking an interaction between SDF-1 and its receptor CXCR4 inhibited MOMC generation, further confirming SDF-1′s critical role in this process. Finally, circulating MOMC precursors were found to reside in the CD14^+^CXCR4^high^ cell population.

**Conclusion:**

The interaction of SDF-1 with CXCR4 is essential for the transformation of circulating monocytes into MOMCs.

## Introduction

Circulating CD14^+^ monocytes, which are heterogeneous in terms of surface markers, phagocytic capacity, and differentiation potential, are committed precursors in transit from the bone marrow to their ultimate site of activity [1]. Until recently, it was believed that monocytes could only differentiate into cells with phagocytic capacity such as macrophages, and dendritic cells [1]–[3]. However, there is growing evidence that circulating monocytes can differentiate into a variety of cell types in addition to phagocytes [4]–[8]. We previously identified a peripheral blood-derived cell population, termed monocyte-derived multipotential cells (MOMCs), that have a fibroblast-like morphology in culture and a unique phenotype positive for CD14, CD45, CD34, and type I collagen [9]. This population originates from circulating CD14^+^ monocytes, and contains primitive cells that can differentiate into cells with the typical phenotypes and functions of mesenchymal cells, neurons, and endothelium *in vitro* [8]–[12]. We recently showed that the intracranial delivery of MOMCs enhances functional recovery in a rat model for ischemic stroke [13]. On the other hand, MOMCs derived from patients with systemic sclerosis were functionally altered and deficient in their ability to differentiate into endothelial cells, either *in vitro* or *in vivo* [14]. These findings raised the possibility that circulating monocytes may contribute to tissue remodeling and regeneration through MOMC differentiation in physiologic and pathogenic states.

MOMCs are obtained with a 7-day culture of peripheral blood mononuclear cells (PBMCs) on fibronectin-coated plastic plates with 10% fetal bovine serum (FBS) as the only source of growth factors. Our previous research indicated that to generate MOMCs from circulating CD14^+^ monocytes *in vivo*, the monocytes are required to bind to fibronectin’s RGD domain via α_5_β_1_ integrin on the monocytes’ cell surface, and are required to be exposed to soluble factor(s) derived from the circulating CD14^−^ cell fraction [9], [15]. However, the details of the molecular factors involved in this process remain unknown. In this study, we examined molecular factors involved in MOMC differentiation by focusing on soluble factor(s) derived from circulating CD14^−^ cells.

## Materials and Methods

### Cell Preparations

PBMCs were isolated from heparinized venous blood from 10 healthy volunteers, aged 22–38, using Lymphoprep (Axis-Shield PoC AS, Oslo, Norway) density-gradient centrifugation. In some experiments, 3.8% sodium citrate was used as an anticoagulant instead of heparin. Since the PBMC fraction was contaminated with a large number of platelets, we removed platelets from the PBMC fraction by MACS column separation using anti-CD61 monoclonal antibody (mAb)-coupled magnetic beads (Miltenyi Biotech, Bergisch Gladbach, Germany). The platelet-depleted PBMCs were then divided into CD14^+^ monocytes and CD14^−^ PBMCs by MACS column separation using anti-CD14 mAb-coupled magnetic beads (Miltenyi Biotech). In some instances, the CD14^+^ monocytes were separated into two distinct populations based on high or low CXCR4 expression, by fluorescence-activated cell sorting. Specifically, CD14^+^ monocytes were incubated with PE-conjugated anti-CXCR4 mAb (BD Biosciences, San Diego, CA) or FITC-conjugated anti-CD11a mAb (Beckman-Coulter, Fullerton, CA) in combination with PC5-conjugated anti-CD14 mAb (Miltenyi Biotech), and were sorted with a FACS® Calibur system. The CD14^+^ cells with CXCR4 levels in the top and bottom 40% were used as CD14^+^CXCR4^high^ and CD14^+^CXCR4^low^ cells, respectively. CD14^+^CD11a^+^ cells were also isolated as a control for the sorting procedure.

Platelets were prepared from platelet-rich plasma by a gel-filtration method described elsewhere [16], with modifications to avoid activating the platelets during the isolation procedure. Briefly, sterilized Sepharose CL-2B gel (GE Healthcare Biosciences AB, Uppsala, Sweden) in phosphate buffer (0.1 M NaH_2_PO_4_⋅2H_2_O, adjusted to pH 7 with NaOH) was washed and equilibrated with Tyrodes buffer (140 mM NaCl, 3 mM KCl, 1 mM MgCl_2_, 16.62 mM NaHCO_3_, pH 7.4, 5.5 mM glucose, and 10 mM HEPES) containing 0.5% bovine serum albumin (BSA) (Sigma, St. Luis, MO). After packing the Sepharose beads into a column, Tyrodes buffer containing 0.5% BSA was replaced with serum-free low-glucose Dulbecco’s modified Eagle’s medium (DMEM; Sigma). Platelet-rich plasma was prepared from peripheral blood by centrifugation (120 g for 10 minutes) and gently applied to the Sepharose column. Platelets were then collected in clouded drips before the plasma proteins exited the column.

The concentration of isolated cells was determined by counting the cells under an inverted microscope (IX81: Olympus, Tokyo, Japan). The protocol of this study was approved by the Ethics Committee of Keio University School of Medicine. All blood samples were obtained from subjects who had given written informed consent.

### Preparation of Platelet-Conditioned Medium

Platelet-conditioned medium was prepared from autologous platelets by several methods. Platelets (4×10^7^/mL) in low-glucose DMEM containing 10% FBS (JRH Bioscience, Lenexa, KS) were cultured on plastic plates coated with fibronectin (Sigma) for 24 hours, and supernatants recovered by centrifugation (180 g for 10 minutes) were used directly as platelet-conditioned medium. Alternatively, platelets (2.5×10^8^/mL) in serum-free low-glucose DMEM were stimulated with one of the following platelet agonists: thrombin (1 U/mL; Mochida Pharmaceutical Co., Ltd., Tokyo, Japan), thrombin receptor activation peptide (SFLLRN; 50 μM), type I collagen (4 μg/mL), adenosine diphosphate (ADP; 400 μM), ristocetin (15 mg/mL), or epinephrine (50 μM) (Sigma) at 37°C for 15 minutes. Supernatants prepared by centrifugation and diluted 10 times with low-glucose DMEM containing 10% FBS were also used as platelet-conditioned medium. In some experiments, medium conditioned by thrombin-treated platelets was fractionated using an Ultrafree-MC Centrifugal Filter® (Millipore, Bedford, MA) with a 30-kDa nominal MW limit, resulting in media enriched in either proteins with a molecular weight (MW) greater than 30 kDa, or with proteins with an MW less than 30 kDa.

### Cultures for Generating MOMCs

In the original method, MOMCs are generated by culturing PBMCs (2×10^6^/mL) for 7 days in low-glucose DMEM containing 10% FBS on fibronectin-coated plastic plates, without any additional growth factors [9]. To evaluate the cell populations and soluble factors required to transform circulating monocytes into MOMCs, sorted CD14^+^ monocytes (2×10^5^/mL) were cultured on fibronectin-coated plastic plates with or without autologous platelets (4×10^7^/mL), CD14^−^ PBMCs (10^6^ cells/mL), or unfractionated or fractionated platelet-conditioned medium. In addition, CD14^+^ monocytes were cultured on fibronectin-coated plastic plates in low-glucose DMEM containing 10% FBS, in the presence or absence of serial concentrations of the following: interleukin (IL)-7, epidermal growth factor (EGF), basic fibroblast growth factor (bFGF), transforming growth factor-β (TGF-β), platelet-derived growth factor (PDGF)-AA, PDGF-AB, IL-8, growth-related oncogene-α (GRO-α), epithelial cell-derived neutrophil-activating peptide 78 (ENA78), thymus and activation-regulated chemokine (TARC), stromal cell-derived factor (SDF)-1, platelet factor 4 (PF4), regulated upon activation, normal T cell expressed and secreted (RANTES), macrophage inflammatory protein-1α (MIP-1α), monocyte chemotactic protein-3 (MCP-3), or neutrophil-activating peptide 2 (NAP2). All of these cytokines, growth factors, and chemokines were purchased from R&D Systems (Minneapolis, MN, USA). In some instances, AMD 3100 (Sigma) was added to the MOMC generation culture at a final concentration of 0, 1, or 5 ng/mL.

In all cultures, medium containing floating cells was exchanged with fresh low-glucose DMED with 10% FBS on day 3. The number and morphology of adherent cells was assessed under an inverted microscope on day 7. The number of total and spindle-shaped adherent cells in a 1 mm x 1 mm square was counted on 10 randomly selected fields, and the efficacy of adherent cell generation was expressed as the proportion (%) of the number of the cells of interest in the culture compared to those observed in a culture of CD14^+^ monocytes alone, in low-glucose DMEM containing 10% FBS during the entire culture period. In experiments using CD14^+^CXCR4^high^ and CD14^+^CXCR4^low^ cells, the results were expressed in comparison with CD14^+^CD11a^+^ cell cultures. The adherent cells were subsequently recovered with 0.25% trypsin and assayed for cell-surface phenotype and *in vitro* differentiation capacity.

### Flow Cytometric Analysis

After staining with FITC-conjugated anti-CD34, FITC-conjugated anti-CD11a or PE-conjugated anti-CXCR4 mAb in combination with PC5-conjugated anti-CD14 mAb, cells were analyzed on a FACS® Calibur flow cytometer using CellQuest software (BD Biosciences). Viable cells were identified by gating based on forward and side scatters, and data were shown as logarithmic dot-plots or histograms.

### Capacity for *In Vitro* Differentiation into Mesenchymal and Endothelial Lineages

Adherent cells obtained in various MOMC generation cultures were replated on fibronectin-coated chamber slides (BD Biosciences) in high-glucose DMEM containing 10% FBS, and were grown to semi-confluence. The cells were then cultured under conditions known to induce the differentiation of MOMCs into mesenchymal and endothelial lineages [9], [12]. MOMCs cultured for 7 days under a mesenchymal-induction condition, as described previously [9], were analyzed for mesenchymal lineage-specific transcription factors, such as Cbfa1 for osteogenesis, Sox-9 for chondrogenesis, and peroxisome proliferation-activated receptor γ (PPARγ) for adipogenesis. For these analyses, the cells were incubated with goat anti-Cbfa1 or anti-Sox-9 polyclonal antibodies, or mouse anti-PPARγ mAb (Santa Cruz Biotechnology, Santa Cruz, CA), followed by AlexaFluor® 568 anti-goat or -mouse IgG antibodies (Molecular Probes, Eugene, OR). The cells were incubated with FITC-conjugated mouse anti-CD45 mAb (Dako Carpinteria, CA) and observed under a fluorescence microscope (IX82; Olympus, Tokyo, Japan).

In some experiments, mesenchymal induction cultures were maintained for 3 to 4 weeks, and differentiation into functional osteoblasts, chondroblasts, and adipocytes was detected by alizarin red staining, immunostaining for type II collagen, and oil red O staining, respectively [9]. The differentiation of MOMCs into the endothelial lineage was evaluated by fluorescent staining with mouse anti-endothelial nitric oxide synthase (eNOS) mAb (BD Biosciences) or rabbit anti-Tie-2 polyclonal antibodies (Santa Cruz Biotechnology), followed by incubation with AlexaFluor® 568 anti-mouse or anti-rabbit IgG antibodies (Molecular Probes) [12]. Negative controls were slides incubated with isotype-matched mouse or rabbit mAb to an irrelevant antigen, instead of the primary antibody. Nuclei were counter-stained with 4′, 6-diamidino-2-phenylindole, dihydrochloride (DAPI).

### Statistical Analysis

All continuous values are shown as the mean ± standard deviation (SD). Comparisons between two groups were tested for statistical significance using the non-parametric Mann-Whitney *U* test.

## Results

### Identification of Circulating CD14^−^ cells Required For Generating MOMCs

**Figure 1:**
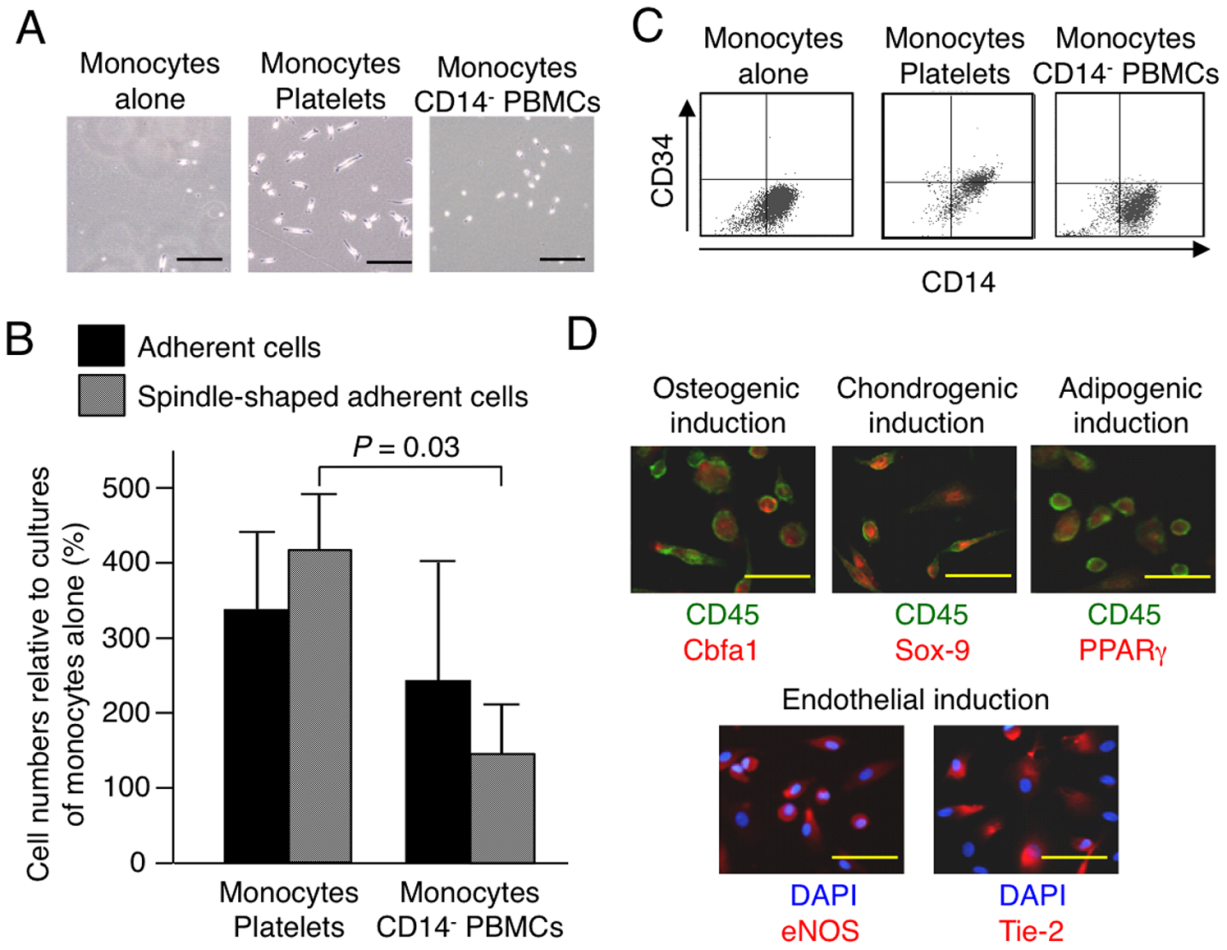
Platelets are required for the generation of MOMCs. CD14^+^ monocytes were cultured on fibronectin with platelets or platelet-depleted CD14^−^ PBMCs, or on fibronectin alone. (**A**) Morphology of adherent cells on culture day 7. Bars: 200 µm. (**B**) The total number of cells and the number of spindle-shaped adherent cells generated are expressed in proportion (%) to those generated by monocyte culture alone. The results shown are the mean and SD of three independent experiments. (**C**) Cell-surface CD14 and CD34 expression on the cultured adherent cells, as analyzed by flow cytometry. (**D**) The multidifferentiation potential of adherent cells obtained by culturing monocytes and platelets. Cells treated for osteogenic, chondrogenic, adipogenic, and endothelial induction for 1 week were analyzed by immunohistochemical staining for Cbfa1, Sox-9, or PPARγ (red) in combination with CD45 (green), or for eNOS or Tie-2 (red) in combination with DAPI (blue), and were observed under a fluorescence microscope. Representative results of three independent experiments are shown. Bars: 50 µm.

We previously reported that to generate MOMCs, circulating CD14^+^ monocytes are required to bind to fibronectin and be exposed to peripheral blood CD14^−^ cells [9]. To identify the circulating CD14^−^ cells required for generating MOMCs, we first evaluated the potential role of platelets that contaminated the PBMC fraction isolated by Lymphoprep density-gradient centrifugation. We prepared CD14^+^ monocytes and CD14^−^ PBMCs from the PBMC fraction after removing the contaminating platelets by negative selection with anti-CD61 mAb-coupled magnetic beads. In addition, circulating platelets were isolated using a gel-filtration method to avoid activating the platelets during the separation procedure. Next, we set up MOMC generation cultures in which CD14^+^ monocytes were cultured alone, with platelets, or with platelet-depleted CD14^−^ PBMCs on fibronectin-coated plastic plates. More adherent cells appeared in the cultures with platelets or CD14^−^ PBMCs than in the culture with monocytes alone (Figure 1A and B). Adherent cells with a fibroblastic morphology typical of MOMCs predominated in the cultures with monocytes and platelets, but were rarely detected in those with monocytes alone or with monocytes and CD14^−^ PBMCs. Flow cytometric analysis showed that CD34 was expressed by the adherent CD14^+^ cells obtained from the cultures of monocytes and platelets, but not by those from cultures of monocytes alone or monocytes with CD14^−^ PBMCs (Figure 1C).

The adherent cells obtained in these cultures were placed in differentiation induction cultures to assess their ability to differentiate into mesenchymal and endothelial lineages. As shown in Figure 1D, the adherent cells obtained in cultures with monocytes and platelets showed nuclear expression of the lineage-specific transcription factors Cbfa1, Sox-9, and PPARγ together with CD45, upon 7-day osteogenic, chondrogenic, and adipogenic induction treatment, respectively, and expression of the endothelial markers eNOS and Tie-2 upon endothelial induction treatment. In addition, functional osteoblasts, chondrocytes, and adipocytes were detected after 3 weeks of osteogenic, chondrogenic, and adipogenic induction treatment of adherent cells obtained in cultures with monocytes and platelets (data not shown). Adherent cells obtained in cultures of monocytes alone or monocytes plus CD14^−^ PBMCs lacked the capacity to differentiate into mesenchymal or endothelial lineages. Concordant findings were obtained from peripheral blood cells derived from three additional donors. Adherent cells obtained in cultures of monocytes and platelets on fibronectin had typical MOMC characteristics, including fibroblast-like morphology, CD34 expression, and the potential to differentiate toward mesenchymal and endothelial lineages [9], indicating that platelets are the primary circulating cell population contributing to the transformation of circulating CD14^+^ monocytes into MOMCs.

**Figure 2:**
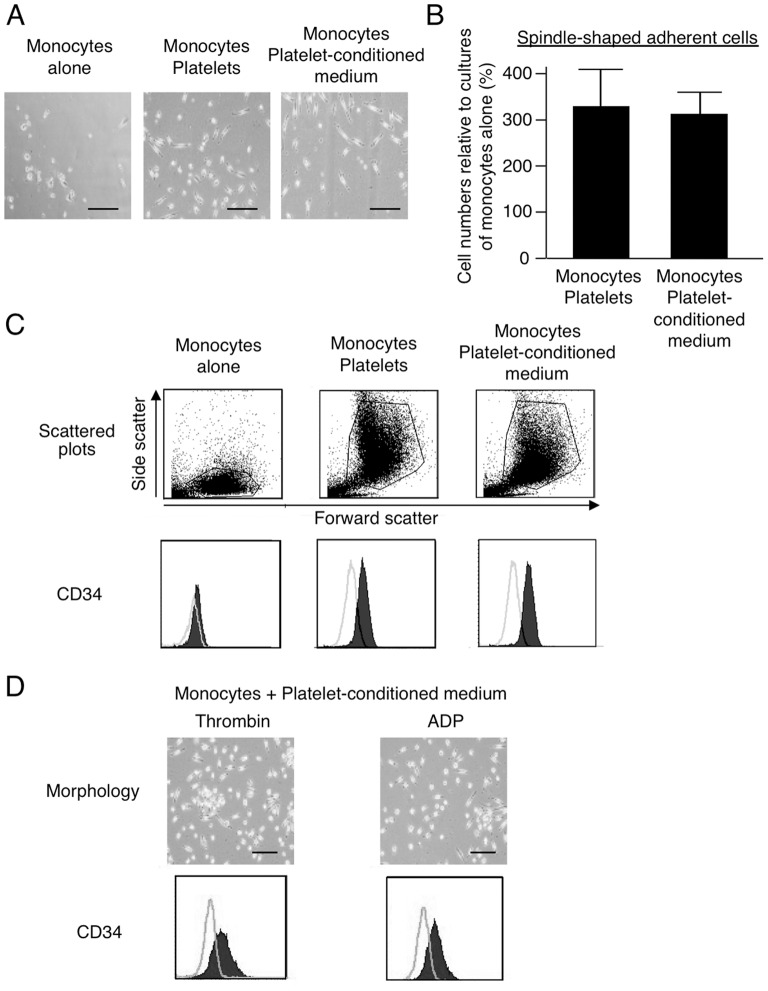
Soluble factor(s) released from activated platelets are required for MOMC generation. CD14^+^ monocytes were cultured alone or in combination with platelets or platelet-conditioned medium on fibronectin. (**A**) Morphology of adherent cells on culture day 7. Bars: 200 µm. (**B**) Spindle-shaped adherent cells generated in the indicated culture, expressed as a proportion (%) of those generated in a culture of monocytes alone. Results show the mean and SD of three independent experiments. (**C**) Scatter plots and surface expression of CD34 on adherent cells, analyzed by flow cytometry. CD34 expression is shown by a closed histogram; open histograms represent staining with isotype-matched control mAb. (**D**) The morphology and CD34 expression of adherent cells obtained from cultures with platelet-conditioned medium prepared by stimulating platelets with thrombin or ADP. Bars: 200 µm. Cell-surface CD34 expression, analyzed by flow cytometry, is shown by closed histograms.

We next examined whether platelet-conditioned medium could be substituted for whole platelets for generating MOMCs. For this purpose, we evaluated MOMC generation by culturing CD14^+^ monocytes with platelets, or with platelet-conditioned medium prepared by culturing platelets on fibronectin. These two cultures yielded adherent cells with fibroblastic morphology with almost the same efficiency (Figure 2A and B). Flow cytometry showed similar scatter distributions and CD34 expression in the adherent cells obtained in these cultures (Figure 2C). The adherent cells obtained in these cultures were able to differentiate into mesenchymal and endothelial lineages according to the induction treatment (data not shown). Thus, MOMCs could be generated in the presence of soluble factors released by platelets that have been activated by exposure to fibronectin.

We further evaluated the MOMC-generation capacity of platelet-conditioned media prepared by stimulation with a variety of platelet agonists, including thrombin, thrombin receptor activation peptide, type I collagen, ADP, ristocetin, and epinephrine. All the platelet agonists induced the release of MOMC-induction factor(s), which promoted the generation of MOMCs in cultures of CD14^+^ monocytes on fibronectin. The representative morphology and flow cytometry analysis of CD34 expression in the adherent cells obtained in cultures containing platelet-conditioned medium prepared by stimulation with thrombin or ADP are shown in Figure 2D. Consistent results were obtained from independent experiments using peripheral blood cells from three additional donors. These results indicated that, to generate MOMCs, circulating CD14^+^ monocytes are required to be exposed to soluble factor(s) released from activated platelets, irrespective of the agonistic stimulation.

### Screening and Identification of a MOMC Differentiation Factor

**Figure 3:**
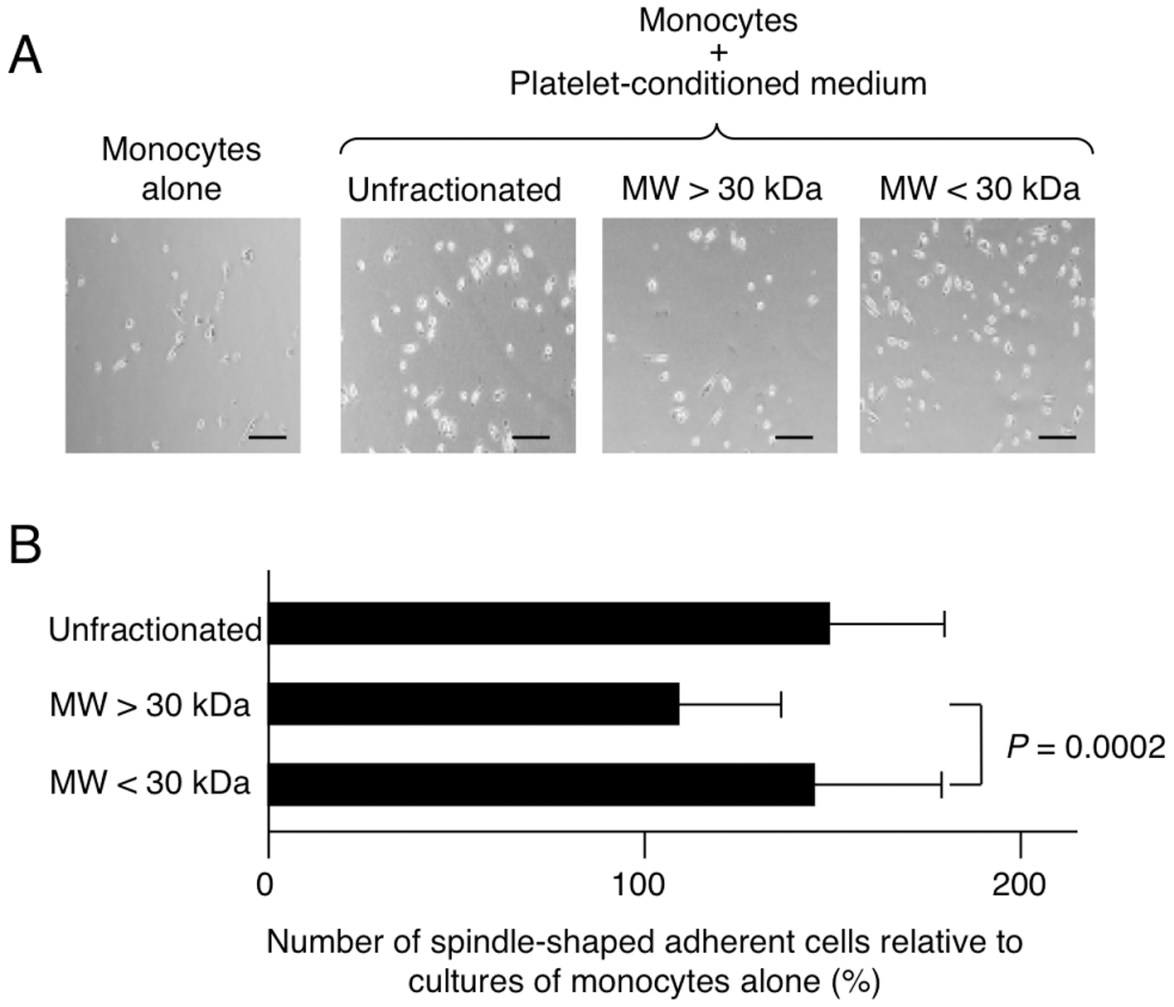
The MOMC generation activity resides in the MW <30-kDa fraction of platelet-conditioned medium. CD14^+^ monocytes were cultured on fibronectin with or without unfractionated or fractionated platelet-conditioned medium prepared by stimulating platelets with thrombin. (**A**) Adherent cell morphology on culture day 7. Bars: 200 µm. (**B**) The generation of spindle-shaped adherent cells in the indicated cultures, expressed as a proportion (%) of those generated by culturing monocytes alone. Results show the mean and SD of three independent experiments.

To screen for MOMC differentiation factor(s), the capacity to transform CD14^+^ monocytes into MOMCs was evaluated by the appearance of adherent cells with fibroblast-like morphology and CD34 expression, observed by flow cytometry, and the ability to differentiate into various lineages. CD14^+^ monocytes were cultured with fibronectin in the presence of thrombin-stimulated platelet-conditioned media fractionated based on a protein MW greater or smaller than 30 kDa (Figure 3). The capacity to generate MOMCs was retained in the platelet-condition medium enriched for proteins with a MW <30 kDa. Concordant results were obtained from five donors.

**Figure 4:**
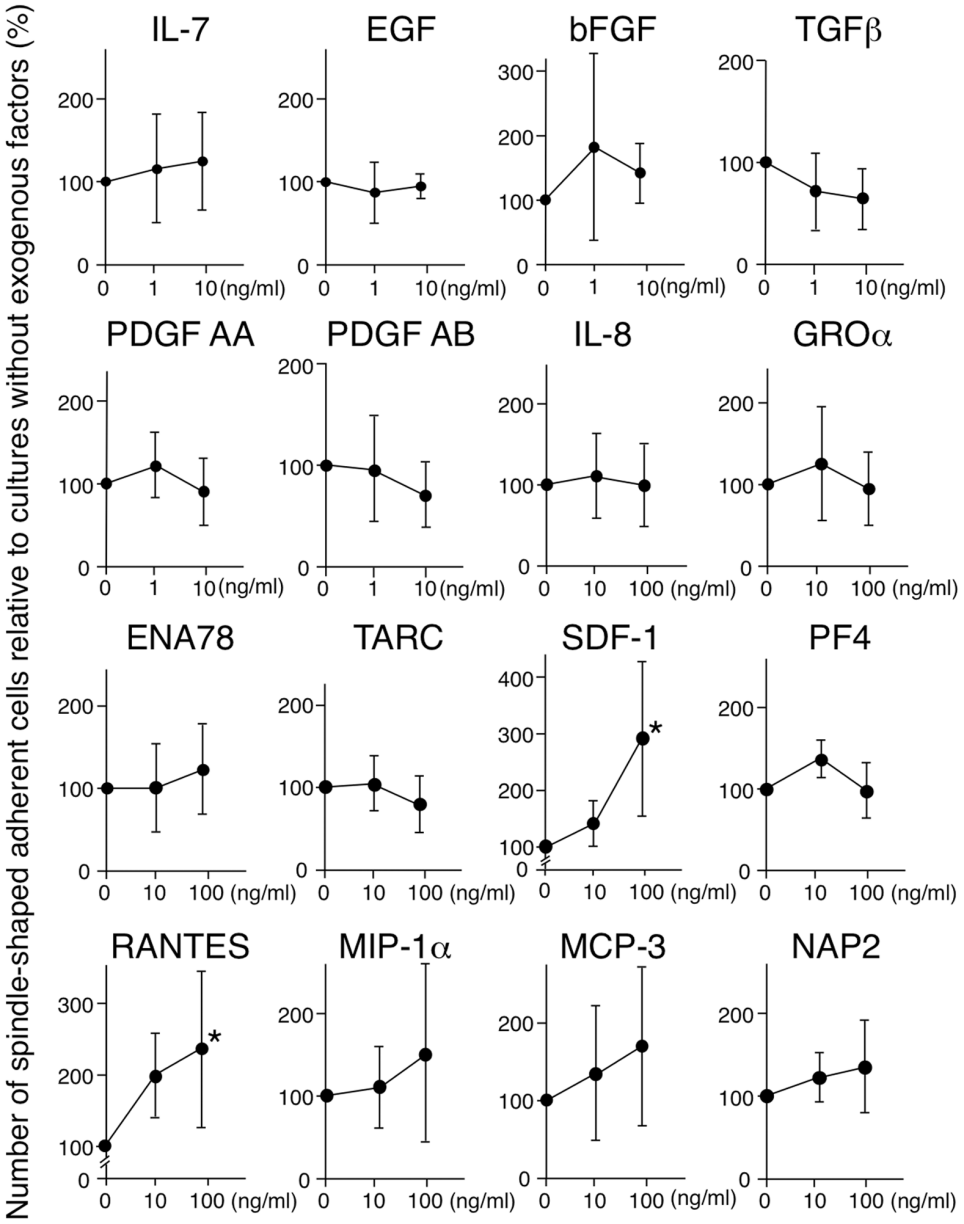
Screening of MOMC differentiation factor(s): 16 candidate cytokines, growth factors, and chemokines. CD14^+^ monocytes were cultured on fibronectin with or without serial concentrations of various soluble factors released by activated platelets and having a MW <30 kDa. The generation of spindle-shaped adherent cells was expressed as a proportion (%) of those generated by culturing monocytes alone. Results show the mean and SD of five independent experiments. *P<0.05, compared with the culture without exogenous factors. IL, interleukin; EGF, epidermal growth factor; bFGF, basic fibroblast growth factor; TGFβ, transforming growth factor-β; PDGF, platelet-derived growth factor; GROα, growth-related oncogene-α; ENA78, epithelial cell-derived neutrophil-activating peptide 78; TARC, thymus and activation-regulated chemokine; SDF-1, stromal cell-derived factor-1; PF4, platelet factor 4; RANTES, regulated upon activation, normal T cell expressed and secreted; MIP-1α, macrophage inflammatory protein-1α; MCP-3, monocyte chemotactic protein-3; and NAP2, neutrophil-activating peptide 2.

We then selected 16 candidate soluble factors required for MOMC generation, based on (i) being released from platelets upon activation and (ii) having a MW <30 kDa. These candidate factors included IL-7, EGF, bFGF, TGF-β, PDGF-AA, PDGF-AB, IL-8, GRO-α, ENA78, TARC, SDF-1, PF4, RANTES, MIP-1α, MCP-3, and NAP2. Serial concentrations of these candidate cytokines, growth factors, and chemokines were added to cultures of CD14^+^ monocytes on fibronectin derived from five donors, and the generation of MOMCs was evaluated (Figure 4). SDF-1 and RANTES were selected as potent factors that promoted the generation of spindle-like adherent cells in a dose-dependent manner (*P*<0.05 for both comparisons); bFGF, MIP-1α, and MCP-3 also promoted the appearance of adherent cells to some extent. Among the adherent cells generated in cultures with these candidate soluble factors, CD34 expression was detected in the monocytes cultured with SDF-1, but not in those cultured with RANTES, bFGF, MIP-1α, or MCP-3.

**Figure 5:**
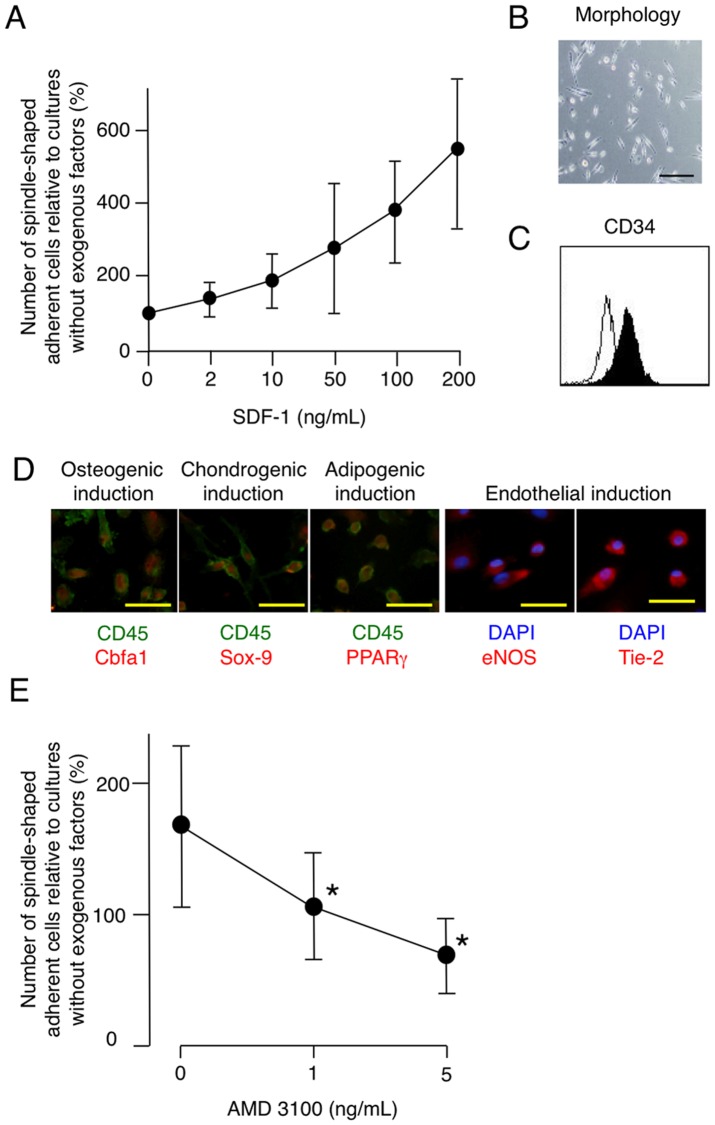
SDF-1 is required for generating MOMCs. CD14^+^ monocytes were cultured on fibronectin in the presence of serial concentrations of SDF-1. (**A**) The generation of spindle-shaped adherent cells, expressed as a proportion (%) of those generated from culturing monocytes alone. The results shown are the mean and SD of 10 independent experiments. (**B**) Morphology of adherent cells obtained in a culture with 100 ng/ml SDF-1. Bars: 200 µm. (**C**) Cell-surface CD34 expressed on adherent cells obtained in a culture with 100 ng/ml SDF-1, as analyzed by flow cytometry. Closed histograms indicate CD34 expression; open histograms represent staining with isotype-matched control mAb. (**D**) Multidifferentiation potential of adherent cells obtained in a culture with 100 ng/ml SDF-1. Cells treated for osteogenic, chondrogenic, adipogenic, and endothelial induction for 1 week were analyzed by immunohistochemical staining for Cbfa1, Sox-9, or PPARγ (red) in combination with CD45 (green), or for eNOS or Tie-2 (red) in combination with DAPI (blue), and were observed under a fluorescence microscope. Representative results of 3 independent experiments are shown. Bars: 50 µm. (**E**) AMD 3100, a CXCR4 antagonist, suppressed the generation of MOMCs. Circulating monocytes were cultured with platelet-conditioned medium on fibronectin in the presence of 0, 1, or 5 ng/ml AMD 3100. Results are expressed as a proportion (%) of the number of spindle-shaped adherent cells obtained in the culture of monocytes alone. *P<0.05, compared with culturing without AMD 3100.

To confirm that SDF-1 was capable of inducing MOMCs, CD14^+^ monocytes from 10 independent donors were cultured on fibronectin in the presence of serial concentrations of SDF-1. SDF-1 increased the number of adherent cells with fibroblast-like morphology, in a dose-dependent manner (Figure 5A and B). Adherent cells generated in the presence of SDF-1 expressed CD34 (Figure 5C) and had multidifferentiation potential toward the osteogenic, chondrogenic, adipogenic, and endothelial lineages (Figure 5D). In addition, functional osteoblasts, chondrocytes, and adipocytes were successfully generated with 4-week mesenchymal induction treatment (data not shown). SDF-1 is a chemotactic factor for a variety of hematopoietic-lineage cells expressing the SDF-1 receptor CXCR4. To examine how blocking the interaction between SDF-1 and CXCR4 would affect MOMC generation, monocyte cultures on fibronectin with thrombin-treated platelet-conditioned medium were prepared in the presence of serial concentrations of AMD 3100, a CXCR4 antagonist. As shown in Figure 5E, AMD 3100 suppressed the generation of spindle-shaped adherent cells in a dose-dependent fashion.

These findings together indicate that the platelet-derived factor SDF-1 is required for generating MOMCs. However, in MOMC generation cultures consisting of CD14^+^ monocytes, fibronectin, and SDF-1, at least 5% FBS was required in the media to generate MOMCs (results not shown), indicating that other factor(s) in the serum were also required.

### Effect of the Circulating CD14^+^ Monocytes’ CXCR4 Expression Level on the MOMC Differentiation Capacity

**Figure 6:**
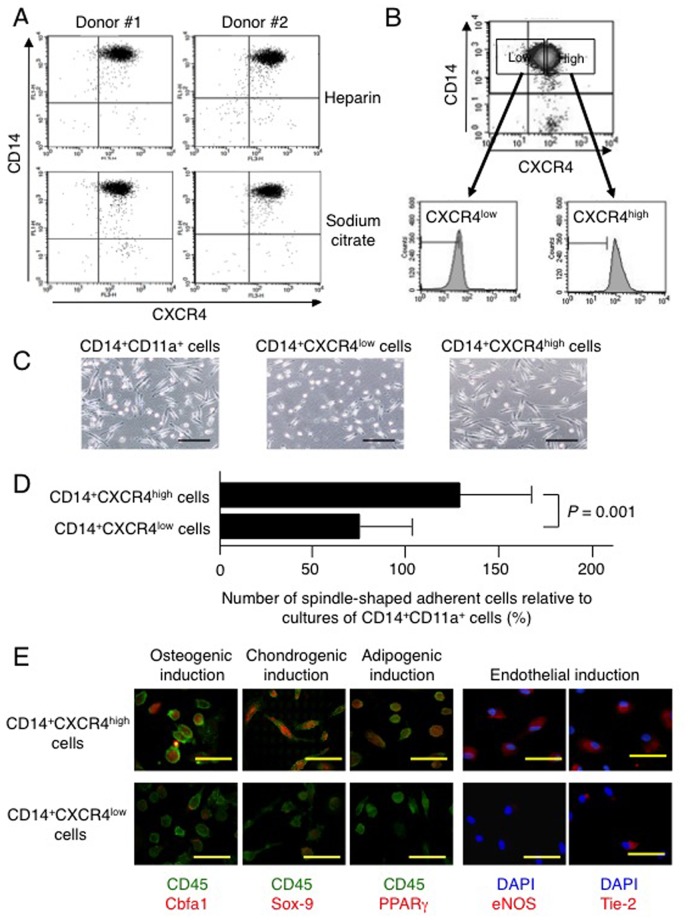
MOMC precursors are enriched in CD14^+^CXCR4^high^ cells. (**A**) Dot-plot analysis of expression of CD14 and CXCR4 in CD14^+^ monocytes derived from 2 independent donors. Peripheral blood samples were anticoagulated with heparin or sodium citrate immediately after blood sampling. (**B**) Dot-plot analysis of CXCR4 expression in CD14^+^ cells, gated by CXCR4^high^ and CXCR4^low^ cells for fluorescent-activated cell sorting. Cell-surface CXCR4 on CXCR4^high^ and CXCR4^low^ cells is shown as histograms. (**C**) Morphology of adherent cells obtained from CD14^+^CD11a^+^, CD14^+^CXCR4^high^, or CD14^+^CXCR4^low^ cells cultured on fibronectin with platelet-conditioned medium. Bars: 200 µm. (**D**) The generation of spindle-shaped adherent cells, expressed as a proportion (%) of those generated by culturing CD14^+^CD11a^+^ cells. Results show the mean and SD of three independent experiments. (**F**) Multidifferentiation potential of cells obtained by culturing CD14^+^CXCR4^high^ and CD14^+^CXCR4^low^ cells. Cells treated for osteogenic, chondrogenic, adipogenic, and endothelial induction for 1 week were analyzed by immunohistochemical staining for Cbfa1, Sox-9, or PPARγ (red) in combination with CD45 (green), or for eNOS or Tie-2 (red) in combination with DAPI (blue), and were observed under a fluorescence microscope. Representative results of 3 independent experiments are shown. Bars: 50 µm.

Nearly all the CD14^+^ monocytes in circulation express CXCR4 on their surface, but the expression level is highly variable (Figure 6A). The expression level of CXCR4 was almost the same between CD14^+^ monocytes derived from peripheral blood samples anticoagulated with heparin and sodium citrate. To evaluate how the level of CXCR4 expressed by circulating CD14^+^ monocytes affects their capacity to differentiate into MOMCs**,** we prepared CXCR4^high^ and CXCR4^low^ monocytes using fluorescent-activated cell sorting (Figure 6B). Flow cytometry revealed that these two cell populations expressed CXCR4 differently. The CD14^+^CXCR4^high^ and CD14^+^CXCR4^low^ cells were then cultured on fibronectin in the presence of thrombin-treated platelet-condition medium. Since nearly all the CD14^+^ monocytes in circulation express CD11a, CD14^+^CD11a^+^ cells prepared by fluorescent-activated cell sorting were used as a control. After 7 days of culture, spindle-shaped adherent cells appeared more frequently in cultures of CD14^+^CXCR4^high^ cells than in cultures of CD14^+^CXCR4^low^ cells (Figure 6C and D), although adherent cells from these cultures expressed CD34 similarly (data not shown). Adherent cells obtained from the CD14^+^CXCR4^high^ cell cultures expressed Cbfa1, Sox-9, and PPARγ upon 7-day osteogenic, chondrogenic, and adipogenic induction cultures, respectively, and expressed eNOS and Tie-2 in endothelial induction cultures (Figure 6E). In contrast, these lineage-specific transcription factors and endothelial proteins were scarcely detectable in the adherent cells cultured from CD14^+^CXCR4^low^ cells. MOMCs were also generated by culturing CD14^+^CXCR4^high^ cells in the presence of SDF-1 instead of platelet-conditioned medium (data not shown). These results together indicate that MOMC precursors are abundant in the circulating CD14^+^CXCR4^high^ cell fraction.

## Discussion

Using a stepwise approach, this study successfully identified SDF-1 as a soluble factor required for transforming circulating CD14^+^ monocytes into MOMCs. First, we found that platelets played a critical role in generating MOMCs from circulating CD14^+^ monocytes. Subsequently, soluble factors with a MW <30 kDa released from activated platelets were shown to contain MOMC differentiation factor(s). The screening of candidate platelet-derived soluble factors identified SDF-1 as a factor required for MOMC generation. The critical role of SDF-1 in this process was further confirmed by the finding that blocking the SDF-1-CXCR4 interaction with AMD 3100 inhibited the MOMC generation. Finally, we found that circulating monocytic MOMC precursors were enriched in the CD14^+^CXCR4^high^ cell population. Taken together with our previous findings [15], the transformation of circulating monocytes into MOMCs requires multiple signals through cell-surface α_5_β_1_ integrin and CXCR4. This information should be useful for investigating how circulating CD14^+^ monocytes acquire multidifferentiation potential, and for establishing an optimal culture condition for generating MOMCs for potential use in regenerative medicine.

SDF-1, also designated CXCL12, is a chemotactic factor for such varied cell types as stem cells, progenitor cells, and mature hematopoietic-lineage cells, such as monocytes. During embryogenesis, SDF-1 is critically involved in the development of hematopoietic, nerve, and endothelial tissues through its regulation of tissue progenitor cell migration, homing, and survival [17], [18]. In adult life, the SDF-1-CXCR4 axis is the key factor for stem- and immune-cell trafficking. For example, hematopoietic stem cells migrate from the bone marrow along the SDF-1 concentration gradient and contribute to tissue repair [19], [20]. Moreover, Yamaguchi and colleagues found that SDF-1 released from activated platelets into the microcirculation is functionally involved in recruiting bone marrow-derived endothelial progenitor cells to vascular injury areas [21]. In contrast to its chemotactic activities, we have found that SDF-1 also modulates the cellular phenotype and differentiation potential of circulating CD14^+^ monocytes.

Although relatively little is known about CXCR4′ s downstream signaling cascades, it has been shown that the binding of SDF-1 to CXCR4 in hematopoietic-lineage cells triggers G protein-mediated downstream signaling, including phosphoinositide-3 kinase, protein kinase A, protein kinase C, and mitogen-activated protein kinase, as well as G protein-independent tyrosine phosphorylation of Janus kinase (JAK) 2 and other members of the JAK signal transduction pathway [22]. These effector pathways regulate cell adhesion, locomotion, and chemotaxis, but how these signaling pathways elicit biological effects, such as proliferation and differentiation, is controversial. Given that α_5_β_1_ integrin is also required for generating MOMC [15], interactions between CXCR4 and integrin signals are of potential interest [23]. In this regard, it has been reported that CXCR4 and integrin signals interact with each other and are crucial for retaining progenitor cells in the bone marrow by phosphorylating c-kit [24], a receptor tyrosine kinase that functions in such diverse biological functions as proliferation, survival, and differentiation [25]. To determine the mechanisms underlying the transdifferentiation of circulating monocytes into multipotential cells, we must examine the downstream signaling pathways of CXCR4 and α_5_β_1_ integrin.

Activated platelets are known to release a variety of soluble factors stored in their granules, including cytokines, chemokines, and growth factors [16]. Interestingly, SDF-1 is produced mainly by mesenchymal cells and endothelium, not by platelets or other hematopoietic-lineage cells. Platelets hold substantial amounts of SDF-1 in their granules that exists in a complex with CXCR4, and release SDF-1 when activated by various agonists [26]. Platelets have long been considered to function solely as chief effector cells in hemostasis. However, the growing evidence that soluble mediators released from activated platelets are involved not only in hemostasis, but also in inflammation, innate/adaptive immune responses, and tissue repair indicates that platelets are more multifunctional than previously thought [27].

Circulating monocytes are recruited to extra-vascular injury sites, where they are exposed to inflammatory cytokines, and differentiate into macrophages that take up debris, clean the tissue, and induce an acquired immune response. Since the differentiation of CD14^+^CXCR4^high^ monocytic precursors into MOMCs requires that the monocytes bind to fibronectin and be exposed to SDF-1 released from activated platelets, circulating monocytes may encounter these signals at the injury site. In that case, these external signals would induce specific intracellular signals in the infiltrating monocytic precursors, leading to the acquisition of mesenchymal and endothelial differentiation potentials. The MOMCs would subsequently differentiate into tissue-specific cells in response to organ-specific environmental cues.

Fibrocytes, a population of circulating cells with fibroblast properties, are characterized by a distinctive phenotype positive for CD45, CD34, and type I collagen [28]. MOMCs and fibrocytes in cultures commonly have spindle-shaped morphology and express CD34 and type I collagen, but have several distinct characteristics. Specifically, MOMCs are generated from circulating CD14^+^ monocytes without expression of CD34 [9]: this phenotypic feature is apparently distinct from that of circulating fibrocytes (CD45^+^CD34^+^type I collagen^+^). In addition, fibrocytes are able to self-replicate and expand in long-term cultures [28], whereas MOMCs have limited lifespan at least *in vitro* [8], [9]. Finally, isolation of fibrocytes in PBMC cultures requires either fibronectin or type I collagen [26], but MOMC induction cultures using type I collagen instead of fibronectin fail to generate CD34^+^ cells with multiple differentiation potentials [15]. These findings strongly suggest that circulating MOMC precursors are different from fibrocytes.

In summary, our results indicate that MOMCs are generated from circulating CD14^+^CXCR4^high^ monocytic precursors through their exposure to fibronectin and SDF-1. These findings are helpful for understanding the mechanisms at work in the transdifferentiation of monocytic progenitors into multipotential cells, and the role of these cells in physiologic and pathogenic states.
